# Efficient Suzuki–Miyaura C-C Cross-Couplings Induced by Novel Heterodinuclear Pd-bpydc-Ln Scaffolds

**DOI:** 10.3390/molecules23102435

**Published:** 2018-09-23

**Authors:** Fu Ding, Yanli Li, Pingxuan Yan, Yan Deng, Dongping Wang, Yajing Zhang, Ileana Dragutan, Valerian Dragutan, Kangjun Wang

**Affiliations:** 1 Faculty of Chemical Engineering, Shenyang University of Chemical Technology, Shenyang 110142, China; liyanli0929@163.com (Y.L.); 13942008761@163.com (P.Y.); dengyan509@163.com (Y.D.); dpwang2015@163.com (D.W.); yjzhang2009@163.com (Y.Z.); 2 Institute of Organic Chemistry, Romanian Academy, Spl. Independentei 202B, P.O.Box 35-108, 060023 Bucharest, Romania; idragutan@yahoo.com

**Keywords:** heterobimetallic catalysts, dinuclear complexes, rare-earth metals, Suzuki–Miyaura cross-coupling

## Abstract

An easy access to a series of previously unreported heterodinuclear Pd-Ln compounds, Pd-bpydc-La, Pd-bpydc-Ce and Pd-bpydc-Nd (bpydc = 2,2′-bipyridine-5,5′-dicarboxylate) has been developed. The Pd-Ln hybrid networks were effectively applied as catalysts in Suzuki–Miyaura C-C cross-coupling reactions of 4-bromoanisole and 4-bromobenzonitrile with phenylboronic acid, under mild conditions. A systematic investigation revealed Pd-bpydc-Nd as the most active catalyst. In all cases, reaction yields varied with the base, catalyst loading and substantially augmented with temperature (from 30 to 60 °C). Substituent effects were operative when changing from 4-bromoanisole to 4-bromobenzonitrile. The key role played by the lanthanides, aromatic substrate and base, in modulating the Pd-catalytic cycle has been highlighted. Importantly, the new catalysts proved to be stable in air and vs. functionalities and are quite efficient in Suzuki–Miyaura carbon-carbon bond formation conducted in protic solvents.

## 1. Introduction

Palladium-catalyzed C-C bond construction [1,2,3,4,5,6,7,8] that makes use of readily available substrates and proceeds with high chemoselectivity has established itself as a flexible synthetic protocol for obtaining substituted arene and heteroarene derivatives [9,10,11,12,13] (e.g., symmetrical or unsymmetrical biaryls) [14]. This synthesis approach has revolutionized the production of advanced materials [15,16,17,18], pharmaceuticals [19,20,21], agrochemicals [21,22], liquid crystals [23], and natural or biologically active compounds [24,25,26,27,28], etc. Numerous attractive strategies have been developed to address the ample scope of this catalytic process including the utilization of palladium nanoparticles [29,30,31,32,33,34,35], or palladium immobilized on magnetic nanoparticles [36] and natural supports [37], use of nucleophilic carbene ligands (mostly NHCs) [38,39,40,41,42,43,44,45,46], Schiff bases [47,48], water-soluble ligands like poly(ethylene glycol)-functionalized N-heterocyclic carbenes [49], thiourea [50] or phosphines [51,52], induction by microwave (MW) acceleration [53,54], use of “greener” solvents, such as water [55,56,57,58,59,60,61,62,63,64,65] (activated by MW [66,67] or in catalysis under micellar conditions [68] for enhancing the solubility of the aromatic halide), water–DMF [69] or ionic liquids [70]. The reaction has long been the subject of vast research in transition-metal catalysis. Various transition metal complexes [71,72,73,74,75,76,77,78,79] (e.g., Ni, Au, Co, Cu, etc., as such or combined in pairs as heterobimetallic catalysts), or nonconventional Pd(II)-complexes with intricate organic ligands containing S or Se have been intensively explored [80,81]. Efficient transition metal-free catalysts have also been reported for Suzuki–Miyaura cross-coupling reactions [82].

Significantly, within the last few years, a new generation of heterogeneous catalysts based on metal–organic frameworks (MOFs) with an Ln-Pd heterobimetallic construction, has been developed and effectively applied to C-C cross-couplings. In such multifunctional configurations, the lanthanide nodes are interconnected by appropriate organic linkers to form rigid porous networks which attach PdCl_2_, prone to catalyze C-C bond formation. Thus, Jin and coworkers [83] ingeniously combined Pd nitrophilic metal cations, which coordinate diimine groups from the 2,2′-bipyridine-5,5′-dicarboxylate (bpydc) linker, with the Ln oxophilic rare earth metals (Ln = Sm, Eu, Gd or Tb) to build-up MOFs displaying a good heterogeneous catalytic activity for C-C cross-coupling reactions. With Ln(bpydc)PdCl_2_ embedded MOFs they performed the Suzuki–Miyaura reaction of selected aryl halides with arylboronic acid in toluene at 95 °C reporting appreciable yields in biaryls (82–97% yields, 4 h). More recently, we have developed a novel set of air- and water-stable heterobimetallic coordination complexes [Ln_2_Pd_3_(BPDC)_2_(HBPDC)_2_(*μ*_2_-O)Cl_4_(H_2_O)_6_·nH_2_O]*_m_* (Ln = Pr, Gd, Tb) binding the rare earth elements through the carboxylate groups of the heteroleptic organic ligand, 2,2′-bipyridine-4,4′-dicarboxylic acid and coordinating the nitrophilic Pd cations to the diimine groups of the difunctional organic linker [84]. By properly engineering the Pd network through the inclusion of lanthanides by intermediacy of a totally different organic vector, the newly created hybrid materials exhibited a reasonably robust and stable structural pattern with enhanced physical and chemical features which is dependent on the nature of the lanthanide incorporated. This type of catalyst demonstrated a high performance in eco-friendly and cost-effective Suzuki–Miyaura, Heck and Sonogashira reactions. Subsequently, we have extended this concept to the synthesis of heterobimetallic coordination complexes, [Ln_2_Pd_2_(BPDC)_5/2_(H_2_O)_2_·4H_2_O]*_n_* (Ln = Nd, Sm, Eu, Dy) employing the above bifunctional organic linker 2,2′-bipyridine-4,4′-dicarboxylic acid to associate Pd ions with rare earth elements [85]. The new class of Ln/Pd heterobimetallic coordination polymers offered a user-friendly methodology for application as catalysts in Heck and Suzuki–Miyaura cross-couplings under green reaction conditions (DMF-H_2_O, 1:1). In both chemical transformations, the new class of complexes displayed an excellent catalytic activity (yields of 99% in Heck reaction and 98% in Suzuki–Miyaura reaction) as compared to state of the art. Applying an alternative metallolig and strategy for MOF construction [86,87], a new set of heterobimetallic Pd/Ln MOFs with Sm, Eu, Tb, Dy, as lanthanide nodes, interconnected by a distinct bifunctional organic linker, 1,1′-di(*p*-carboxybenzyl)-2,2′-diimidazole, has been prepared [88]. This novel class of MOFs showed excellent stability in air and water and displayed high catalytic activity in Suzuki–Miyaura reactions conducted both homogeneously and heterogeneously, in neat water, ethanol or water–ethanol. Phase switchability was demonstrated when changing from water (homogeneous reaction) to ethanol (heterogeneous reaction) with beneficial consequences for practical applications. Such complexes were found to be pH-responsive, in a reversible way, enabling convenient recovery from acidic water solutions in view of recycling, as well as for environmentally friendly, final separation of metal residues.

**Scheme 1:**
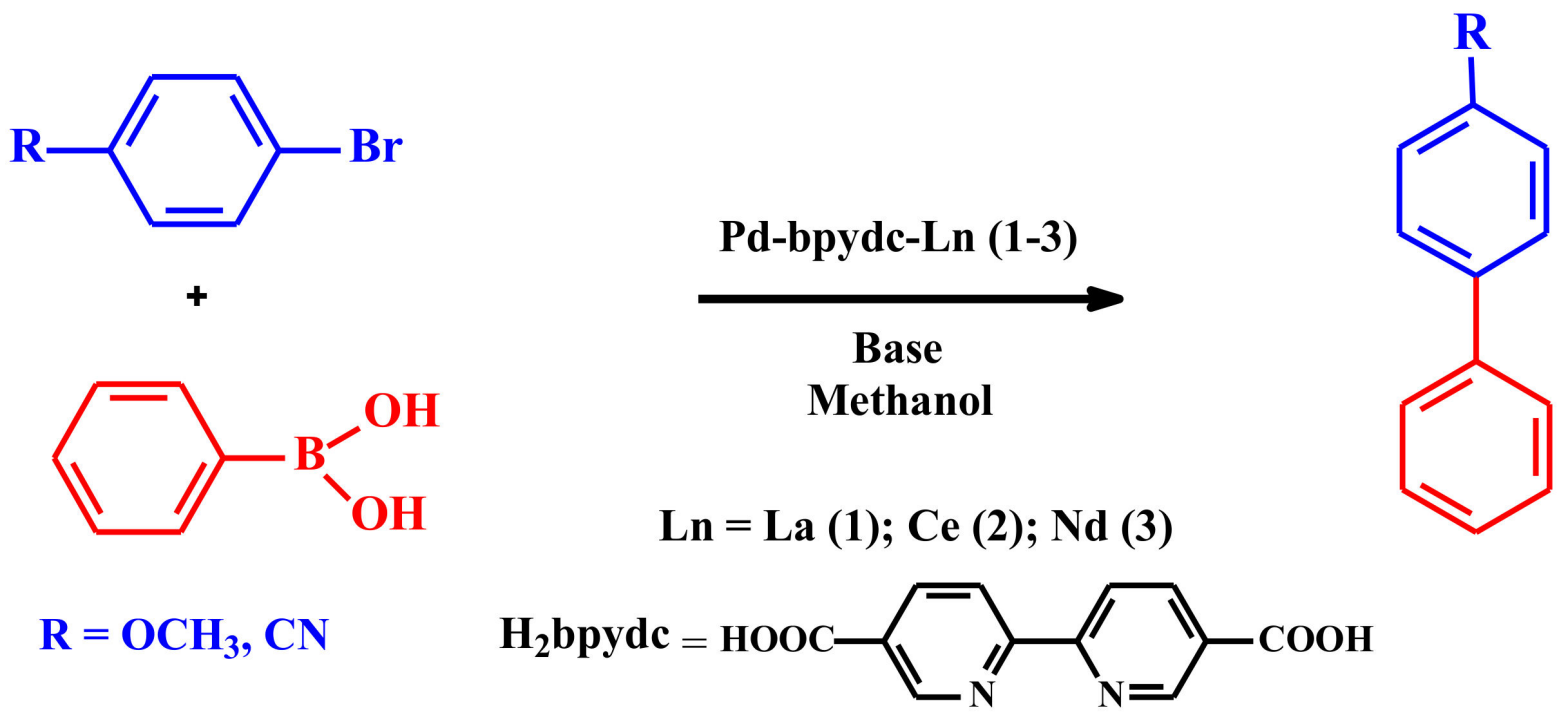
The Suzuki–Miyaura cross-couplings of 4-bromoanisole and 4-bromobenzonitrile with phenylboronic acid promoted by Pd-bpydc-Ln catalysts (**1**–**3**).

With the aim of disclosing new catalytic abilities of rare-earth elements associated with palladium when they are incorporated within heterodinuclear Pd networks by means of 2,2′-bipyridine-5,5′-dicarboxylate (bpydc) as the heteroleptic organic linker, we have synthesized three novel dinuclear Pd-Ln MOF-type catalysts [Pd-bpydc-La (**1**), Pd-bpydc-Ce (**2**), Pd-bpydc-Nd (**3**)] by a simple one-pot procedure and successfully applied them in Suzuki–Miyaura reaction. We took advantage of the oxophilic propensity of the lanthanides to properly bind to the carboxylate groups of the bifunctional organic ligand to create a rigid network tethering Pd chloride at its nucleophilic nitrogen sites. Additionally, the electronic properties of the dipodal organic linker, 2,2′-bipyridine-5,5′-dicarboxylate, will influence the charge transfer between lanthanide and palladium active centers, tailoring, thus, the catalytic properties of the metal–organic framework. The final aim of these investigations lies in the evaluation of the catalytic properties of compounds **1**–**3** in heterogeneous cross-coupling of two model aryl bromides, i.e., 4-bromoanisole and 4-bromobenzonitrile, with phenylboronic acid, in methanol as a solvent, to give the corresponding biphenyl derivatives (Scheme 1).

## 2. Experimental Section

### 2.1. Materials and Synthesis Methodology

All reagents and solvents employed were of commercial grade, purchased from the suitable suppliers and were used without further purification. Powder XRD patterns were recorded with CuKα radiation by using a Bruker D8 Advance X-ray diffractometer. Elemental analyses were performed on a Perkin-Elmer elemental analyzer. Conversions were determined using Agilent 7890 GC-MSD. IR spectra were measured using a Nicolet IR-470 spectrometer for samples in KBr pellets. 

### 2.2. Synthesis and Characterization of Pd-bpydc-Ln (***1***–***3***).

The heteronuclear bimetallic palladium compounds **1**–**3** have been synthesized in a one-pot procedure, according to the previously published methodology [88,89,90]. A mixture of K_2_PdCl_4_ (0.29 mmol), 2,2′-bipyridine-5,5′-dicarboxylic acid (0.29 mmol), Ln(NO_3_)_3_·6H_2_O (0.18 mmol) and water (10 mL) was stirred for 20 min in open air. The mixture was then transferred to a 23 mL Teflon reactor and kept at 100 °C for 24 h under autogenous pressure, thereafter, cooled to room temperature at a rate of 5 °C/h. The resulted yellow crystals were characterized by elemental analysis, powder XRD and infrared spectroscopy (KBr). Anal. Calcd. for C_24_ H_35_N_4_O_19_Cl_4_La_1_Pd_2_ (**1**): C, 24.49; H, 3.00; N, 4.76. Found: C, 24.41; H, 3.08; N, 4.80. IR (KBr)(cm^−1^): 3445, 3062, 1694, 1612, 1592, 1555, 1410, 1389, 1294, 1257, 1143, 1054, 858, 835, 778, 699, 661, 599, 415. Anal. Calcd for C_24_H_35_N_4_O_19_Ce_1_Cl_4_Pd_2_ (**2**): C, 24.46; H, 2.99; N, 4.75. Found: C, 24.38; H, 3.10; N, 4.71. IR (KBr)(cm^−1^): 3448, 3061, 1698, 1613, 1557, 1392, 1296, 1142, 1057, 1037, 858, 893, 776, 700, 661, 527, 419. Anal. Calcd. for C_24_H_35_N_4_O_19_C_l4_Nd_1_Pd_2_ (**3**): C, 24.38; H, 2.98; N, 4.74. Found: C, 24.30; H, 3.10; N, 4.81. IR (KBr)(cm^−1^): 3458, 3071, 1711, 1623, 1567, 1402, 1302, 1161, 1065, 1042, 861, 888, 779, 706, 683, 568, 421. X-Ray analysis indicated that compounds **1–3** are isostructural and crystallize in a triclinic crystal system with space group *P*-1. Powder X-ray diffraction analyses of compounds **1**–**3** have been performed at room temperature. The patterns for **1**–**3** are in good agreement with the calculated data obtained from the single-crystal structures, confirming that the purities equal those of the single crystal samples. 

### 2.3. General Procedure for Suzuki–Miyaura Reaction

A mixture of 4-bromoanisole (0.5 mmol) or 4-bromobenzonitrile (0.5 mmol), phenylboronic acid (0.6 mmol), specified base (0.5 mmol), methanol (1 mL), 0.2–0.5 mol% of catalyst was stirred at 30 °C or 60 °C in air. The progress of the reaction was monitored by withdrawing samples periodically which were analyzed by gas chromatography–mass spectrometry (GC-MS). GC-calculated yields were based on the amount of 4-bromoanisole or 4-bromobenzonitrile employed. At the end of the reaction, the catalyst was separated by simple filtration. The mixture was extracted with diethyl ether (20 mL), washed with water and dried over anhydrous Na_2_SO_4_. The solvent from the extract was completely removed with a rotary evaporator to obtain the product, which was further purified by column chromatography on silica gel. All coupling products were identified by Agilent 7890A-5975C GC-MS and ^1^H NMR spectroscopy.

## 3. Results and Discussion

With the new set of Pd-Ln compounds in hand, we focused first on the optimization studies of the cross-coupling reaction between 4-bromoanisole and phenylboronic acid as special model substrates (Scheme 1) striving to identify the best catalytic system and reaction conditions. On this line, catalysts **1**–**3** and various combinations of temperatures, reaction times and catalyst/substrate ratios were investigated (Table 1). 

**Figure 1:**
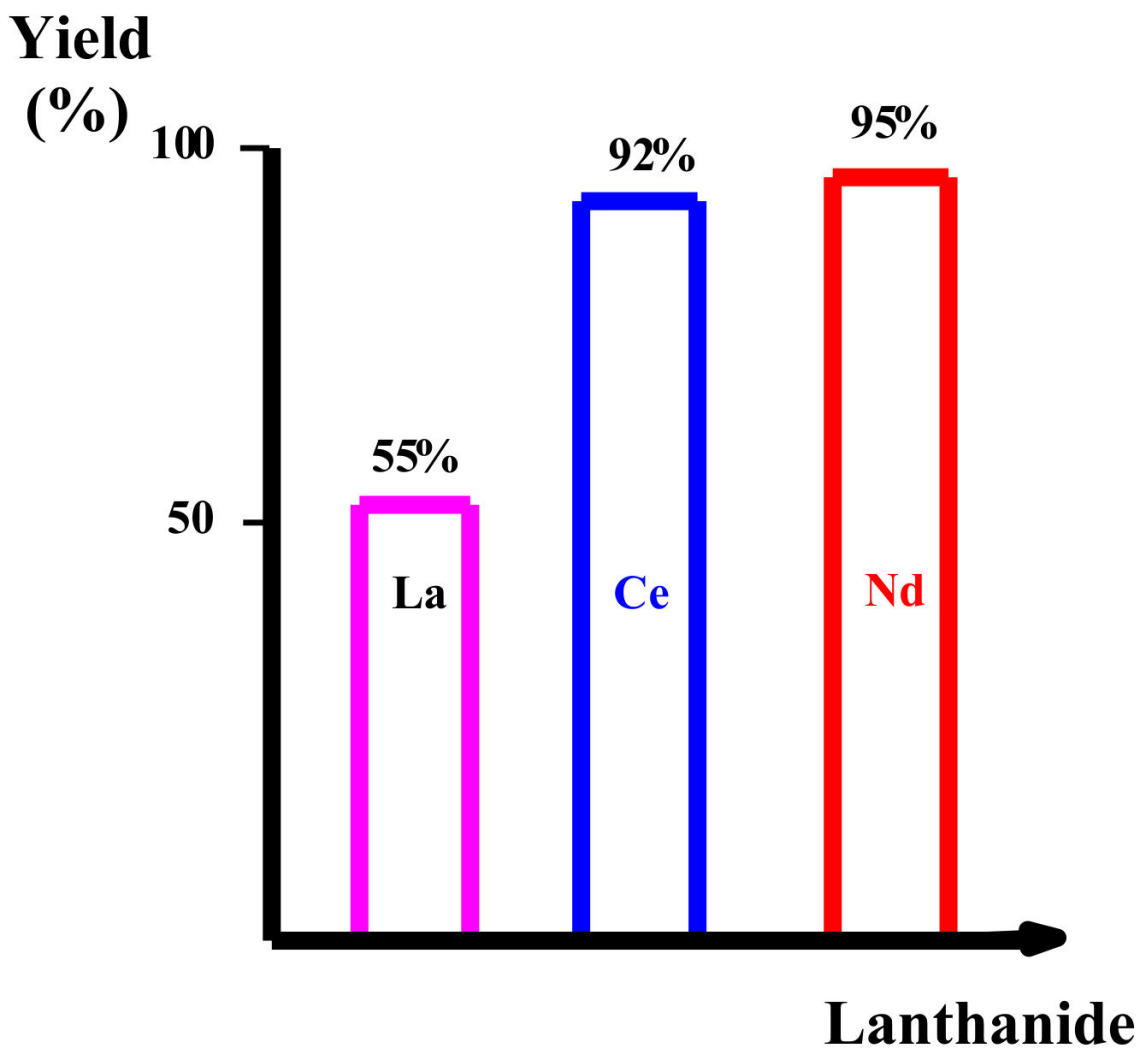
Product yields in Suzuki–Miyaura reaction of 4-bromoanisole with phenylboronic acid catalyzed by **1**–**3**.

For properly surveying and comparing the catalytic performance of our three Pd-bpydc-Ln compounds (**1**–**3**), we used a range of bases and methanol to ensure good solubility of the reaction partners and intermediates (best for the sodium *tert*-butoxide–methanol pair). Catalytic runs were carried out in the 30–60 °C temperature range, with catalyst/substrate molar ratios corresponding to different catalyst loadings (varying from 0.5 mol % to 0.2 mol %). Notably, low catalyst loadings allowed high yields (93–95%) and conversions (73–99%) to be attained with catalyst **3**, at the optimized reaction time and temperature. For almost all sets of reaction parameters, the recorded conversions and yields follow the order: Pd-bpydc-Nd (**3**) > Pd-bpydc-Ce (**2**) > Pd-bpydc-La (**1**). An increase in the product yield with temperature is the general trend for the three catalysts. This result is in accordance with the fact that palladium-catalyzed cross-coupling in organic solvents is conveniently performed at higher temperature [83]. Under all tested conditions, Pd-bpydc-Nd (**3**) is clearly the best catalyst (Table 1, entries 9–12) affording highest yields (Figure 1) and selectivities. Remarkably, these favorable yields were obtained at 30 °C as well as at 60 °C, which are milder than other currently used temperatures in cross-couplings occurring in common organic solvents. 

**Scheme 2:**
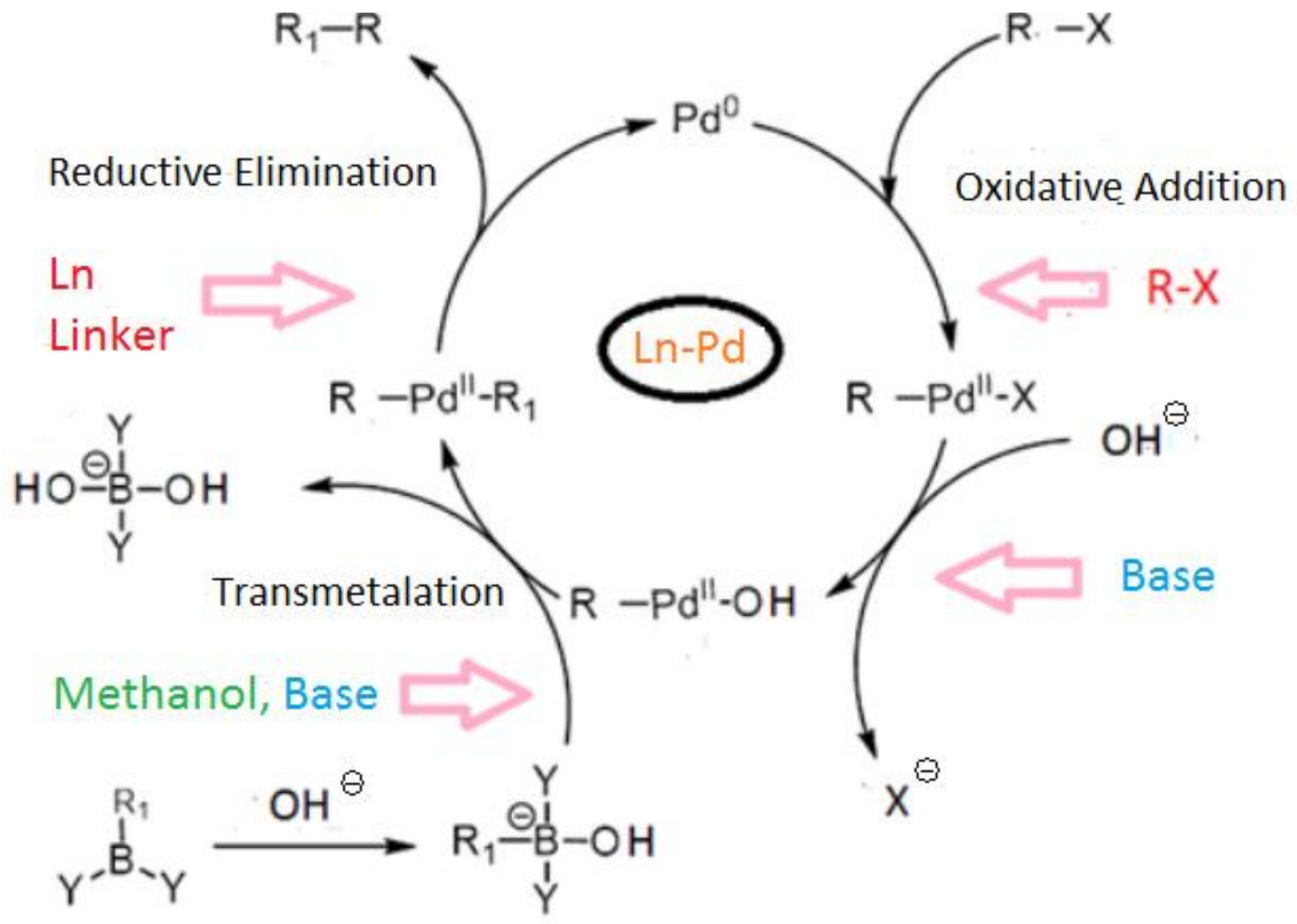
Influence of lanthanides (Ln = La, Ce, Nd), substrate, base and solvent on the Pd catalytic cycle in the Suzuki–Miyaura cross-coupling reaction.

Noteworthy, we found that the nature of the lanthanide highly matters. As will be seen below, the lanthanides play a significant role in tailoring in several ways the catalytic properties of the palladium complexes. Working either at 30 °C or 60 °C and at low catalyst loadings the activity of Pd-bpydc-Nd (**3**) (yield: 95%; Table 1, entry 9 and 11) surpasses that of the previously reported Pd(diimine)Cl_2_ embedded heterobimetallic (Pd-Ln) heterogeneous catalysts [83] which have a related structure but contain another lanthanide (i.e., Ln = Sm). A sharp variation in product yield and catalyst activity with temperature was found for Pd-bpydc-Ce (**2**); this catalyst proved to be highly active at 60 °C, even for a low catalyst loading (Table 1, entry 7 and 8) but displayed lower yields at 30 °C (Table 1, entry 5 and 6) under the employed reaction conditions (8 h reaction time, 0.2 mol % or 0.5 mol % catalyst loading). Most intriguingly, under best conditions catalyst Pd-bpydc-La (**1**) provided only moderate yields (max. 55%, Table 1, entry 3), as compared to Nd and Ce. This totally distinct behavior of the La complex is still not well understood. Since compounds **1**–**3** having as rare-earth elements La, Ce, Nd are isostructural, we assume that the observed dissimilarities in activity should not stem from the crystal structure but originate from the nature of the lanthanides and their mode of coordination into the metal–organic framework. The varying Lewis acid character and electropositivity of the three rare-earth elements may determine the stability and activity of the coordinated Pd sites with consequences on the reaction outcome. To rationalize this different behavior of the Pd-Ln catalysts, we consider that the lanthanides would build a specific electronic environment at the palladium site through the organic linker, as a function of their electropositive propensity and are, therefore, responsible for the activity and stability of the catalyst. Furthermore, the differences in electropositivity and reducing the power of the three lanthanides, La, Ce, and Nd, may affect otherwise the palladium catalytic cycle and particularly the reductive elimination step by favoring the generation of Pd(0) species able to resume a new catalytic cycle (Scheme 2). 

In our case, Nd^3+^ endowed with a small ionic radius and high availability of the f-electrons (Table 2) seems to have a strong influence on the Pd site through charge transfer, mediated by the flexible organic linker, conducive to an enhancement of the reductive elimination step of the Pd catalytic cycle. This process occurs *via* synergistic cooperation between the lanthanide and the aromatic linker in transferring charge density to the nitrogen-Pd bond.

The lanthanides, having multiple coordination abilities and an enhanced oxophilic character, enable the formation of a 3D framework anchoring PdCl_2_ and yielding, thus, a highly active and stable Pd catalyst.

Another significant observation is that low catalyst loadings result in a slight drop in the yield and conversion. Obviously, in our experiments, the yields are consistently higher when the catalyst amount is 0.5 mol %. Moreover, Table 1 convincingly demonstrates that, under the promotion of our dinuclear catalytic systems, the reaction in methanol can lead to advantageous results. The rationale behind the excellent performance in methanol likely lies in the fact that it can dissolve most of the substrate and base ensuring a favorable concentration of these compounds in the reaction medium. Furthermore, methanol is cheap and readily available; however, running catalytic tests in this solvent at more elevated temperatures is hindered by its relatively low boiling point. Gratifyingly, from the above data, Pd-bpydc-Nd catalyst (**3**) in methanol as the solvent is an excellent choice for efficient application in the Suzuki–Miyaura cross-coupling.

Variation of the base (Table 3) showcased that sodium and potassium hydroxide, as well as sodium *tert*-butoxide, are the most appropriate bases affording high yields (Table 3, entries 1, 3 and 5), even at lower catalyst loading (0.2 mol %) and reaction temperature (30 °C).

Interestingly, the other tested inorganic bases (e.g., sodium and potassium carbonates) gave rather good yields proving that the employed bases provide a sustainable outcome in the biaryl product (yields of 92–95%), thus, widening the scope of our catalytic process.

As commonly accepted, an important issue in the Suzuki reaction is the nature of the aryl halide, R-X. In this respect, the above-investigated parameters that affect the reaction course have been conducted on 4-bromoanisole as a model substrate. A different R-X substrate, namely 4-bromobenzonitrile, was additionally examined in cross-couplings with phenylboronic acid (Table 4), under the same reaction conditions as given in Table 3, allowing, thus, an accurate comparison of the two aryl substrates. 

**Figure 2:**
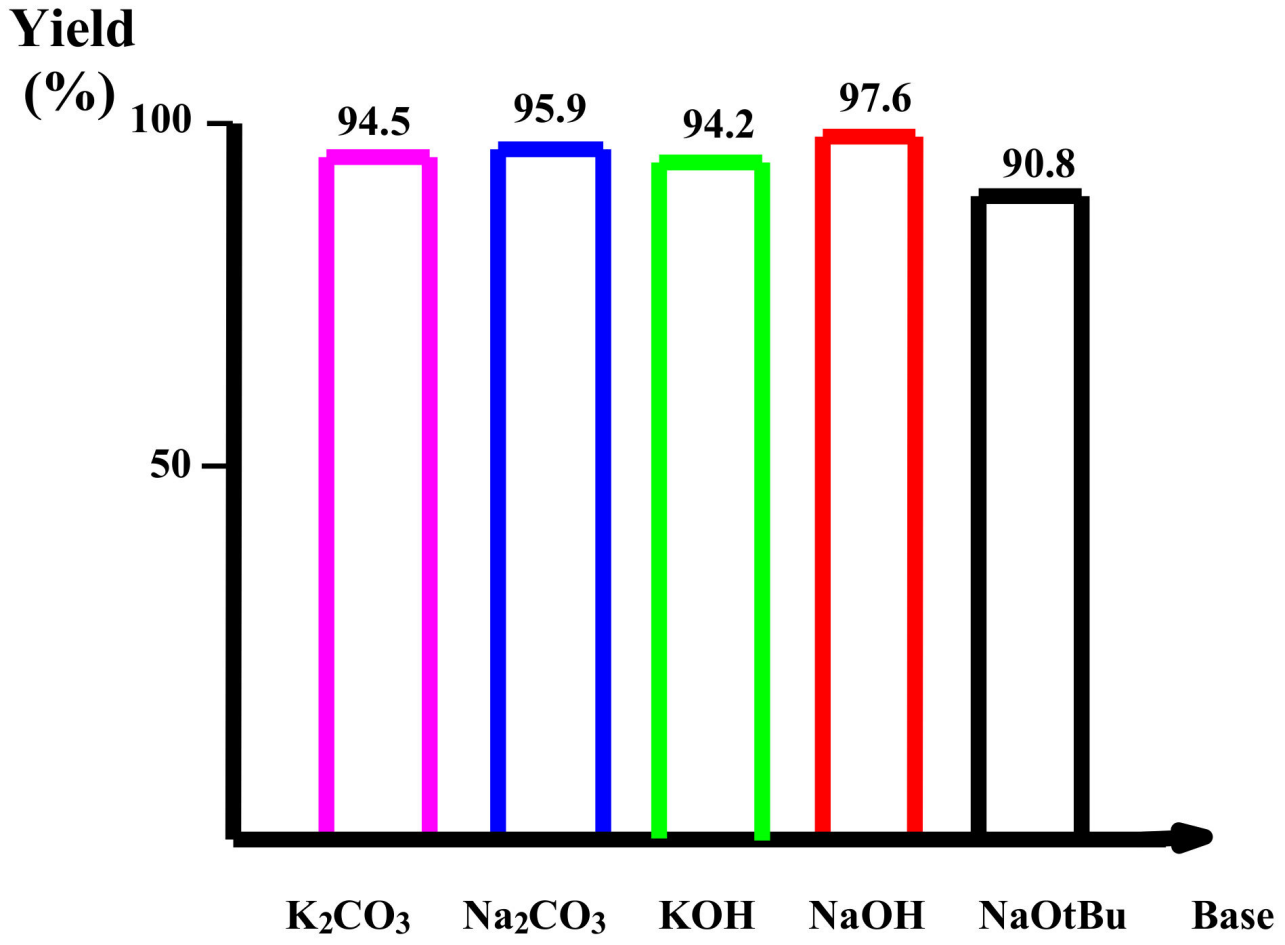
Product yields in Suzuki–Miyaura reaction of 4-bromobenzonitrile with phenylboronic acid catalyzed by **3**.

Studies on the substrate scope unequivocally indicated that, for each base, conversions and yields pertaining to reactions with 4-bromobenzonitrile (Table 4) are superior to those obtained with 4-bromoanisole (Table 3). These results are reasonable taking into account the well-established fact that oxidative addition [Pd(0) to RPd(II)X] is the rate-determining step in the Pd catalytic cycle [5,7,90,93] and, therefore, aryl halides activated by an electron-withdrawing group, as is the case of 4-bromobenzonitrile, are more reactive in the oxidative addition step as compared with substrates bearing electron-donating groups (i.e., 4-bromoanisole). The increase in reactivity of 4-bromobenzonitrile could, thus, explain the obvious difference observed for the carbonates for the two substrates, these weak bases becoming quite effective in C-C couplings with the new aryl bromide (entries 2–4 Table 4 vs. entries 2–4 in Table 3, Figure 2).

Furthermore, the high level of activity maintained by all bases examined by us may be rationalized by considering the same mechanism of action exerted by the base in activating the organoboron compound in the transmetalation step of the Pd catalytic cycle (Scheme 2) [93,94,95]. Inherent differences could be determined by the nature of the counter cations and solubility pattern of the base in the protic medium.

Recyclability of the Pd-bpydc-Nd (**3**) catalyst has been demonstrated in the reaction of 4-bromoanisole with phenylboronic acid, in consecutive runs, working under our mildest conditions. It was found that the catalyst could be reused three times without significant loss of the catalytic activity. Nonetheless, the structure of the catalyst after three runs could not be determined so far and further investigations are to be conducted in this respect.

## 4. Conclusions

In summary, the heterodinuclear compounds, Pd-bpydc-La, Pd-bpydc-Ce, and Pd-bpydc-Nd (bpydc = 2,2′-bipyridine-5,5′-dicarboxylate), synthesized in a one-pot procedure, provided advantageous access to biphenyl derivatives by cross-coupling of aryl bromideswithphenylboronic acid. The attractive feature of our approach lies in the association of palladium, the most active and traditionally employed transition metal for the catalyzed Suzuki–Miyaura reaction, with rare earth metals, i.e., La, Nd or Ce, that together with the organic linker build MOFs with good chemical, thermal and mechanical stability. The beneficial cooperativity between the electropositive Ln ions and the electrophilic Pd ions, providing a distinct electronic environment at the Pd active sites, seems to be favored by the bifunctional organic linker, finally leading to a highly active Pd catalyst.

Both high conversions and selectivities have been attained, under mild conditions. It was found that the Pd-bpydc-Nd catalyst performs best. Use of methanol as the solvent and of alkaline hydroxides or carbonates as the base led to optimum outcomes. Results suggest that the rare earth elements, by influencing the activity of palladium, play an important role in attaining the excellent selectivities observed especially for the combination Pd-Nd. Moreover, this catalyst could be recycled in three consecutive runs. Further efforts to extend the application of the here disclosed catalytic systems to other cross-coupling transformations are underway in our laboratory. 

## Figures and Tables

**Table 1:** Suzuki–Miyaura cross-coupling of 4-bromoanisole with phenylboronic acid in methanol-induced by Pd-bpydc-Ln catalysts **1**–**3**.

Entry	Catalyst	T (°C)	Time (h)	Yield (%) ^a,b,c^
1	Pd-bpydc-La (**1**)	30	8	35.0 ^a^
2		30	8	15.0 ^b^
3		60	4	55.0 ^a^
4		60	4	14.9 ^b^
5	Pd-bpydc-Ce (**2**)	30	8	25.0 ^a^
6		30	8	25.9 ^b^
7		60	4	91.9 ^a^
8		60	4	88.9 ^b^
9	Pd-bpydc-Nd (**3**)	30	8	95.0 ^a^
10		30	8	93.4 ^b^
11		60	4	95.0 ^a^
12		60	4	94.9 ^b^

^a^ Reaction conditions: 4-bromoanisole (0.5 mmol), phenylboronic acid (0.6 mmol), sodium tert-butoxide (0.5 mmol), methanol (1 mL),catalyst (0.5 mol %); ^b^ Catalyst (0.2 mol %).; ^c^ Reaction yield (%) for total conversion determined from the internal standard yield, at the specified reaction time, based on gas chromatography (GC), using hexadecane as internal standard.

**Table 2:** Ln3+ radius (pm) and electron configuration of La, Ce, and Nd.

Lanthanide	La	Ce	Nd
Ln^3+^ radius (pm) ^a^	103	102	98.3
Ln^3+^ electron configuration ^b^	4f^0^	4f^1^	4f^3^

^a^ [91]; ^b^ [92].

**Table 3:** Suzuki–Miyaura cross-coupling of 4-bromoanisole with phenylboronic acid in the presence of Pd-bpydc-Nd (**3**) when various bases are employed ^a^.

Entry	Catalyst	Base	Yield (%) ^b^
1	Pd-bpydc-Nd (**3**)	KOH	95.0
2		K_2_CO_3_	91.9
3		NaOH	92.0
4		Na_2_CO_3_	92.0
5		NaO^t^Bu	95.0

^a^ Reaction conditions: 4-bromoanisole (0.5 mmol), phenylboronic acid (0.6 mmol), base (0.5 mmol), methanol (1 mL); reaction time 8 h; temperature: 30 °C, catalyst: 0.2 mol %. ^b^ Reaction yield (%) for total conversion determined from the internal standard yield, at the specified reaction time, based on the GC, using hexadecane as internal standard.

**Table 4:** Suzuki–Miyaura cross-coupling of 4-bromobenzonitrile with phenylboronic acid in the presence of Pd-bpydc-Nd (**3**) when various bases are used ^a^.

Entry	Catalyst	Base	Yield (%) ^b^
1	Pd-bpydc-Nd (**3**)	KOH	94,2
2		K_2_CO_3_	94.5
3		NaOH	97.6
4		Na_2_CO_3_	95.9
5		NaO^t^Bu	90.8

^a^ Reaction conditions: 4-bromobenzonitrile (0.5 mmol), phenylboronic acid (0.6 mmol), base (0.5 mmol), methanol (1 mL); reaction time 8 h; temperature: 30 °C, catalyst: 0.2 mol %. ^b^ Reaction yield (%) for total conversion determined from the internal standard yield, at the specified reaction time, based on the GC, using hexadecane as internal standard.
